# Basketball’s Improvement in Bone Mineral Density Compared to Other Sports or Free Exercise Practice in Children and Adolescents: A Systematic Review and Meta-Analysis

**DOI:** 10.3390/children12030271

**Published:** 2025-02-24

**Authors:** Cristina Castro-Collado, Francisco Jesus Llorente-Cantarero, Mercedes Gil-Campos, Jose Manuel Jurado-Castro

**Affiliations:** 1 Metabolism and Investigation Unit, Maimonides Biomedical Research Institute of Cordoba (IMIBIC), Reina Sofia University Hospital, University of Cordoba, 14004 Cordoba, Spain; q62cacoc@uco.es (C.C.-C.); juradox@gmail.com (J.M.J.-C.); 2 Department of Specific Didactics, Faculty of Education and Psychology, University of Cordoba, 14004 Cordoba, Spain; 3 CIBEROBN (Physiopathology of Obesity and Nutrition), Institute of Health Carlos III (ISCIII), 28029 Madrid, Spain; 4 Ciencias De La Actividad Física y El Deporte, Escuela Universitaria de Osuna (Centro Adscrito a la Universidad de Sevilla), 41640 Osuna, Spain

**Keywords:** bone health, exercise, bone mass, osteoporosis, childhood

## Abstract

Background: Bone mineral density (BMD) is crucial for bone health, contributing up to 50% of total bone mineral content during childhood and pre-adolescence, with the accumulation of bone mass in youth significantly impacting adult bone health. Physical activity, especially impact exercise, plays a fundamental role in strengthening bones. Objectives: The aim of this meta-analysis was to study the effects of basketball practice on BMD compared to other sports and free activity practice in children and adolescents. Methods: Observational studies were selected up to January 2024. A total of 492 articles were identified, of which 9 met the criteria for inclusion in the meta-analysis. Results: The BMD increase favored the group of basketball players in the total body (MD 0.07; CI 0.04 to 0.09; *p* < 0.001; I^2^ = 93%), upper limbs (MD 0.10; CI 0.008 to 0.12; *p* < 0.001; I^2^ = 96%), and lower limbs (MD 0.05; CI 0.03 to 0.07; *p* < 0.001; I^2^ = 80%). Conclusions: Basketball practice in children and adolescents appears to be one of the most effective sports for enhancing BMD (total body and upper and lower limbs) compared to football, swimming, combat sports, other team sports, such as baseball and volleyball, as well as athletics and gymnastics. The high heterogeneity among studies, largely due to differences in sports, may limit the interpretation of the findings.

## 1. Introduction

During growth stages, bone plays a pivotal role in physical development [1]. These periods involve rapid increases in height and body mass, requiring a substantial accumulation of bone mass to ensure adequate structure, support, and safeguarding of vital organs, such as the brain, heart, or lungs, and prevent chronic bone diseases like osteoporosis, characterized by bone loss and increased fragility [2]. The accumulation of bone mass during these formative years is crucial for shaping bone health in adulthood [3]. Additionally, bone acts as a reservoir for minerals, calculated through bone mineral density (BMD), which is the most common method to assess bone health, notably calcium and phosphorus, releasing them into the bloodstream when necessary [4]. BMD increases significantly during these years, influenced by genetic, nutritional, and environmental factors, including physical activity (PA) [5]. Promoting lifelong bone health entails fostering proper bone development during youth and adolescence and preserving BMD through a balanced diet, regular PA, and comprehensive healthcare practices [6]. Beyond its role in skeletal development, bone health during childhood and adolescence is also closely linked to overall physical performance and injury prevention [4]. Optimal bone mineralization contributes to greater structural integrity, reducing the risk of stress fractures and musculoskeletal injuries commonly observed in young athletes. Moreover, bone strength influences motor skills, coordination, and physical endurance, all of which are essential for sports participation and daily activities [2]. Given that peak bone mass is largely established before adulthood, ensuring proper bone development during these formative years is not only crucial for preventing future conditions, such as osteoporosis, but also for enhancing athletic performance and overall well-being [6].

Research indicates that childhood and pre-adolescence are pivotal phases for BMD development, contributing up to 50% of total body bone mineral content, especially during growth spurts when height increases necessitate a substantial boost in BMD [7]. PA and exercise play a crucial role in modifying and enhancing BMD [6,8]. Engaging in physical activities primarily focused on impact or weight-bearing exercises offers a notable opportunity to enhance BMD [6,9]. In addition, it has shown that those who participated in impact sports at these ages tend to accumulate greater BMD compared to those involved in non-impact sports [9,10]. Moreover, high-impact exercise has also been shown to have a positive effect on non-weight-bearing bones, so there is an overall benefit to all bones in the body in children and adolescents [11]. Basketball appears to be one of the most effective sports for enhancing BMD in children and adolescents due to its combination of high-impact, multidirectional movements and weight-bearing activities [2]. Unlike sports with lower osteogenic potential, such as swimming or cycling, basketball exposes the skeleton to frequent mechanical loads, including jumping, sprinting, and abrupt changes in direction, which stimulate bone remodeling and adaptation. Additionally, compared to other weight-bearing sports like soccer or running, basketball incorporates a greater variety of movement planes and impact magnitudes, leading to more widespread skeletal benefits [10]. This suggests that basketball may play an essential role in maximizing bone mass acquisition during critical growth periods, potentially reducing the risk of osteoporosis and fractures later in life [7]. However, despite its potential advantages, studies evaluating the specific effects of basketball on BMD in children and adolescents remain limited. A more detailed analysis comparing basketball with other sports in this age group is crucial for better understanding its impact and optimizing bone health strategies for young athletes [9].

Regarding sports practice, basketball stands out over other sports by its blend of movements, which encompass varied actions, such as rapid sprints, abrupt halts, or frequent jumps. These movements exert substantial stress on the skeletal system [12,13]. Basketball is a dynamic and non-repetitive sport, which leads to better bone responses to all these movements [14,15]. Previously, it has been suggested that basketball players possess a higher BMD than other sports (free activity, swimming, soccer, or volleyball) [16]. However, this conclusion has been based on collective assessments, including meta-analyses that have combined both children and adults in their samples [16]. Therefore, it is intended to observe if, through practicing this sport, children can achieve better bone health compared to other sports. Nevertheless, considering the characteristics of children and adolescents [17], such as the impact of growth on BMD, it is pertinent to conduct an analysis specifically focusing on the effect of BMD in this age group. The aim of this study was to evaluate the impact of basketball on BMD compared to other sports and free activity in children and adolescents.

## 2. Materials and Methods

A systematic review was conducted, along with a meta-analysis of studies, following the “Preferred Reporting Items for Systematic Reviews and Meta-analysis (PRISMA)” criteria [18]. The PRISMA criteria were assessed by a checklist (Supplementary Material Table S1). The review protocol was registered at the PROSPERO (International Prospective Register of Systematic Reviews), with the registration number CRD42023433664.

### 2.1. Criteria for Considering Studies for Inclusion in the Review

In this systematic review, the search criteria used were to review all clinical trials and observational studies up to January 2024, which were found in the above-mentioned databases, written in English, and published in any country.

Inclusion criteria were defined based on the PI(E)CO(S) system, as follows: P (population): children and adolescents aged 6–18; I(E) (exposure): practice of basketball; (C) (comparison): control group not involved in sports or engaged in other sports; (O) (outcome): BMD; and (S) (study type): observational studies.

Exclusion criteria for studies were as follows: (1) studies in which the participants were adults; (2) studies in which basketball training was not compared; (3) studies in which BMD was not reported accurately or where researchers failed to provide data upon contact; and (4) non-primary studies, such as letters and reviews.

### 2.2. Protocol for Electronic Searching

The bibliographic search was carried out in different databases, including articles published in MEDLINE (Pubmed), WOS (Web of Science), ScienceDirect, and SCOPUS. The keywords used to perform the search were related to “basketball” and “bone mineral density”, adding age ranges by using the words “children” and “adolescents”. More details about the electronic searches are shown in the Supplementary Material (Supplementary Material, File S1).

### 2.3. Study Selection and Data Collection

Two independent reviewers conducted the searches and analyzed the studies (C.C-C and J.M. J-C.). Articles found were coded using the reference manager RefWorks [19]. The data from each article were carefully analyzed to ensure that the interpretation of the data was completely correct. The articles were then screened according to the established inclusion criteria.

The literature search was divided into two phases. In the first phase, articles were selected based on title and abstract, with those not meeting the inclusion criteria being discarded. In the second phase, a thorough analysis of all articles was conducted, extracting information, such as the countries where the studies were conducted, participant ages, sex, the various sports compared in each study, sample details, specific body parts (total body and upper and lower limbs), where BMD measurements were taken, sports practice times, weekly session frequencies, the instruments employed for BMD measurement, and the primary results obtained in each study.

### 2.4. Risk of Bias in Individual Studies

To assess the risk of bias, the Cochrane Risk of Bias 2 (RoB 2) [20] tool was used. This tool evaluates the risk of bias across 5 domains, which are bias arising from the randomization process, bias due to deviation from intended interventions, bias due to missing outcome data, bias in measurement of the outcome, and bias in selection of the reported results, as well as an overall risk of bias. These domains cover all types of bias currently understood to affect RCT and are rated as low risk of bias, some concerns, or high risk of bias.

### 2.5. Statistical Analysis

Data were obtained by considering the sample size, mean, and standard deviation extracted from each study. The random-effects model method was used to measure the effect of the included studies. An analysis of the variation in BMD from basketball training was carried out by comparing it with participating in other sports, as well as children and adolescents engaged in free activity.

An analysis was conducted comparing the basketball group with free activity practice. Moreover, an analysis comparing basketball with each sport, dividing into subgroups between school-age children (9 to 11 years) and adolescents enrolled in higher education (12 to 18 years), was performed. Finally, an analysis was conducted comparing the frequency of basketball training (minutes per week) to that of free activities. The analyses were presented individually, comparing basketball with each sport (free activity, football, volleyball, swimming, judo, karate, and kung fu) to reduce heterogeneity. Data were collected from other sports such as handball, baseball, gymnastics, and athletics, but they could not be included in the meta-analysis due to insufficient studies available for comparative analysis.

The results of this meta-analysis were presented in a forest plot showing mean differences (MDs) with 95% confidence intervals (CI). MDs were employed to interpret the findings to offer a clear and direct measure of the disparity between the study groups and maintain the measurement scales utilized across the included studies. The heterogeneity (I^2^) was calculated by the I^2^ index. The I^2^ index was considered low heterogeneity (0–40%); moderate (30–60%); considerable (50–90%); or substantial (75–100%) [21]. To address heterogeneity, subgroups were created based on body segments and by distinguishing between children and adolescents. This approach also allowed for a better understanding of differences between groups. A funnel plot was conducted (Supplementary Material, File S2).

To carry out the meta-analysis, the Review Manager program version 5.4.1. was used (RevMan, computer program) [22]. The value of *p* < 0.05 in the overall effect Z indicated that there was a significant difference in the analyses. The results are shown as mean values followed by the ‘±’ symbol, representing standard deviation data.

## 3. Results

### 3.1. Studies Selected

**Figure 1:**
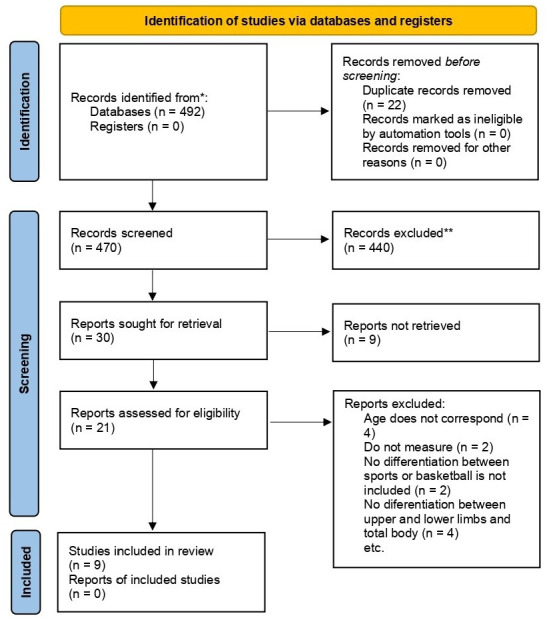
PRISMA flowchart for the selection of relevant studies from databases for this meta-analysis. BMD: bone mineral density.

A flow chart (Figure 1) shows the selection process of the studies included in the meta-analysis. A total of 492 articles were identified from the databases used. A search was carried out with a cut-off date of January 2024. A total of 22 articles were eliminated as duplicates, reducing the number an overall of 470 articles. A total of 449 articles were eliminated for the following reasons: (1) the title and abstract were not related to the search objectives; (2) participants did not meet the inclusion criteria; (3) the studies did not measure BMD; and (4) the participants were not solely involved in basketball. Subsequently, 21 articles were considered eligible for inclusion in the meta-analysis, according to the inclusion criteria. Four articles were excluded because they were conducted in adults or in combination with them; two articles because they did not measure BMD; another two articles because no distinction was made between sports or because basketball was not among the sports; and four articles were excluded because BMD was not measured as distinguishing between lower and upper limbs and total body (Supplementary Material, Table S2). Finally, 9 articles were selected, which met the inclusion criteria for meta-analysis (Figure 1).

### 3.2. Description of Selected Studies

A total of 1344 children and adolescents aged between 9 and 18 years were analyzed in 9 studies. All participants were categorized into distinct groups (children and adolescents). Among them, there were 949 individuals engaged in sports, while 395 participants did not practice any sports.

In the sports group, different subgroups were established in relation to the sport being played: (1) a basketball group, with a total of 234 participants; (2) a swimming group with a total of 180 participants; (3) a football group, with a total of 179 participants; (4) a karate group, with a total of 83 participants; (5) a judo group, with a total of 119 participants; (6) a kung fu group, with a total of 88 participants; and (7) a volleyball group, with a total of 66 participants. Of the selected studies, 6 were conducted in Brazil [12,13,23,24,25,26], one took place in Estonia [9], another took place in Spain [15], and the last one in Tunisia [27] (Table 1).

All participants in the sports group had been practicing regulated exercise for at least 6 months [12,13,24,26]. In two of the previous studies, children and adolescents engaged in free activity were required not to have played sports in the last 3 months [12,26]. Another study included participants who had been practicing sport for approximately 8 years [9], while in another, participants were asked to have been practicing sport for at least one year [27]. One of the studies does not refer to the minimum time spent playing sports, as it was not considered an inclusion criterion [23]. In one of the studies, a period of 3 years was specified [25], and in a third study, minimum training of 8 months was demanded [15].

The training minutes per week ranged from 180 to 1108. In all studies, dual-energy X-ray absorptiometry (DXA) was used to measure BMD, which emits two types of X-rays at different energies through the bone and measures the amount of energy absorbed by the surrounding tissues. This technique provides a detailed image of the bone mineral composition and allows for the calculation of BMD in specific areas of the body [28].

### 3.3. Risk of Bias in Included Studies

Considering the five domains established to analyze the risk of bias, five of them are noteworthy (Supplementary Material, Figure S1). After reading all of the selected articles in depth, none of the articles included in the meta-analysis implemented participant randomization, as each of them was already associated with a specific sport prior to study enrollment, thus being aware of their group affiliation. Data analysis did not commence until after study completion in all instances. In this study, participants who participated in sports were compared to those who did not. However, this does not mean the latter group was inactive or led sedentary lives. Therefore, they were considered to engage in free activity. The authors of these articles did not measure whether children who did not participate in sports engaged in any physical activities during their leisure time.

### 3.4. Effects of the Intervention

To measure the effect of the studies, the increase in BMD in different sports, as well as in free activity, was analyzed. Nine studies were included in the meta-analysis.

#### 3.4.1. Comparative Analysis of BMD in Basketball vs. Free Activity and Other Sports (Total Body and Upper and Lower Limbs)

##### Basketball vs. Free Activity (Total Body and Upper and Lower Limbs)

- Total body

**Figure 2:**
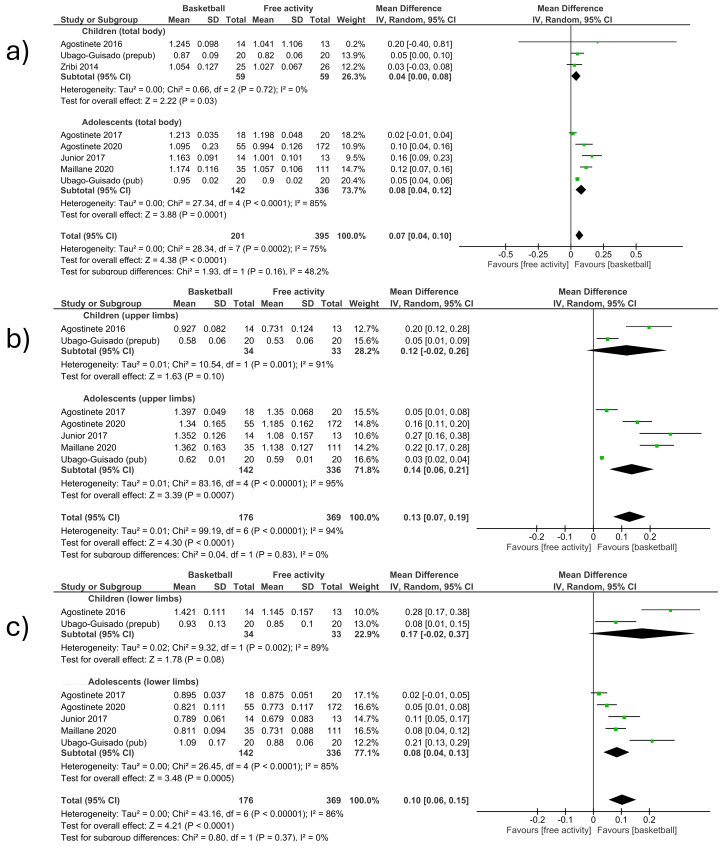
Effect of basketball interventions on BMD increase in children and adolescents compared to other sports and free activity. (**a**) Forest plot illustrating the increase in total body BMD in favor of basketball. (**b**) Forest plot illustrating the increase in upper limb BMD in favor of basketball. (**c**) Forest plot illustrating the increase in lower limb BMD in favor of basketball [12,13,15,23,24,26,27].

The total body BMD was measured in seven studies included in the meta-analysis, comparing basketball vs. free activity (MD (Mean Difference) 0.07; CI (Confidence Interval) as 0.04 to 0.10; *p* < 0.001; I^2^ (I-Squared) = 75%) (Figure 2a). The first section included studies involving children [12,15,27] and observed an increase in BMD in favor of basketball players (MD 0.04; CI 0.00 to 0.08; *p* = 0.03; I^2^ = 0%). The second section included only adolescents [13,15,23,24,26] and observed an increase in BMD in favor of basketball players (MD 0.08; CI 0.04 to 0.12; *p* < 0.001; I^2^ = 85%). The study that had the most weight (20.4%) was located in the second section [15]. No significant differences were observed between the groups of children and adolescents (Figure 2a).

- Upper limbs

The upper limb BMD was measured in six studies included in the meta-analysis, comparing basketball vs. free activity (MD 0.13; CI 0.07 to 0.19; *p* < 0.001; I^2^ = 94%) (Figure 2b). The first section included studies involving children [12,15] and observed an increase in BMD in favor of basketball players (MD 0.12; CI −0.02 to 0.26; *p* = 0.10; I^2^ = 91%). The second section included studies involving adolescents [13,15,23,24,26] and observed an increase in BMD in favor of basketball players (MD 0.14; CI 0.06 to 0.21; *p* < 0.001; I^2^ = 95%). The study that had the most weight (16.6%) was located in the second section [15]. No significant differences were observed between the groups of children and adolescents (Figure 2b).

- Lower limbs

The lower limb BMD was measured in six studies included in the meta-analysis, comparing basketball vs. free activity. (MD 0.10; CI 0.06 to 0.15; *p* < 0.001; I^2^ = 86%) (Figure 2c). The first section included studies involving children [12,15] and observed an increase in BMD in favor of basketball players (MD 0.17; CI −0.02 to 0.37; *p* = 0.08; I^2^ = 89%). The second section included studies involving adolescents [13,15,23,24,26] and observed an increase in BMD in favor of basketball players (MD 0.08; CI 0.04 to 0.13; *p* < 0.001; I^2^ = 85%). The study that had the most weight (17.1%) was located in the second section [23]. No significant differences were observed between the groups of children and adolescents (Figure 2c).

##### Basketball vs. Football (Total Body and Upper and Lower Limbs)

- Total body

**Figure 3:**
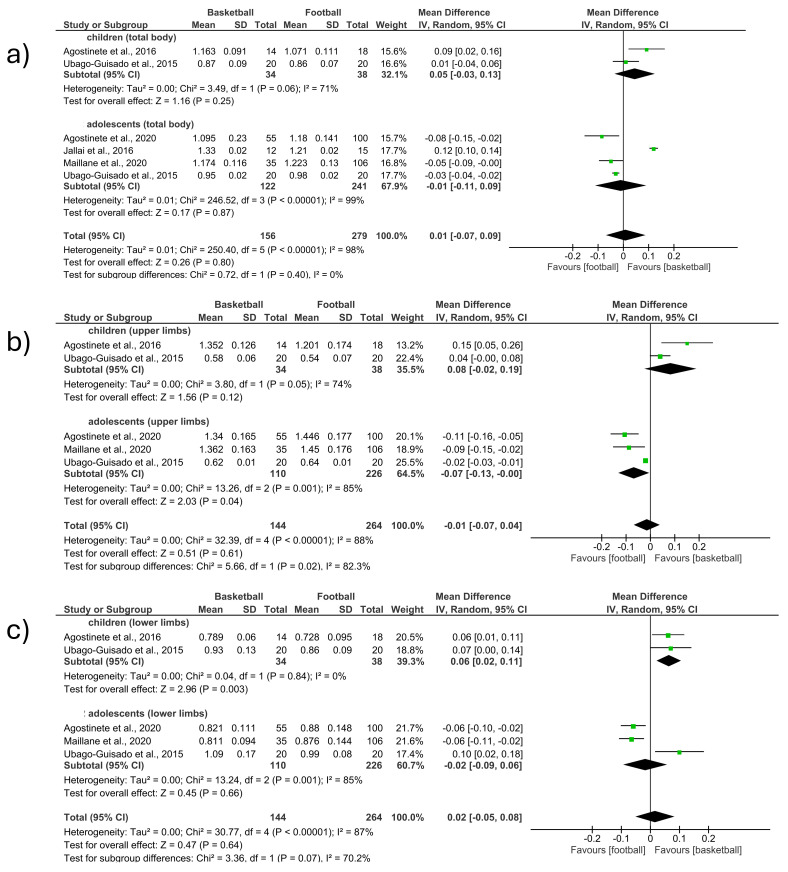
Effect of basketball interventions on BMD increase in children and adolescents compared to football interventions. (**a**) Forest plot illustrating the increase in total body BMD in favor of basketball. (**b**) Forest plot illustrating the increase in upper limb BMD in favor of football. (**c**) Forest plot illustrating the increase in lower limb BMD in favor of basketball [9,12,13,15,24].

The total body BMD was measured in five studies included in the meta-analysis, comparing basketball vs. football (MD 0.01; CI −0.07 to 0.09; *p* = 0.80; I^2^ = 98%) (Figure 3a). The first section included studies involving children [12,15] and observed an increase in BMD in favor of basketball players (MD 0.05; CI −0.03 to 0.13; *p* = 0.25; I^2^ = 71%). The second section included studies involving adolescents [9,13,15,24] and observed an increase in BMD in favor of football players (MD −0.01; CI −0.11 to 0.09; *p* = 0.87; I^2^ = 99%). The studies that had the most weight (17.7%) were located in the second section [9,15]. No significant differences were observed between the groups of children and adolescents (Figure 3a).

- Upper limbs

The upper limb BMD was measured in four studies included in the meta-analysis, comparing basketball vs. football (MD −0.01; CI −0.07 to 0.04; *p* = 0.61; I^2^ = 88%) (Figure 3b). The first section included studies involving children [12,15] and observed an increase in BMD in favor of basketball players (MD 0.08; CI −0.02 to 0.19; *p* = 0.12; I^2^ = 74%). The second section included studies involving adolescents [13,15,24] and observed an increase in BMD in favor of football players (MD −0.07; CI −0.13 to −0.00; *p* = 0.04; I^2^ = 85%). The study that had the most weight (25.5%) was located in the second section [15]. Significant differences were observed between the groups of children and adolescents (*p* = 0.02) (Figure 3b).

- Lower limbs

The lower limb BMD was measured in four studies included in the meta-analysis, comparing basketball vs. football (MD 0.02; CI −0.05 to 0.08; *p* = 0.64; I^2^ = 87%) (Figure 3c). The first section included studies involving children [12,15] and observed an increase in BMD in favor of basketball players (MD 0.06; CI 0.02 to 0.11; *p* = 0.003; I^2^ = 0%). The second section included studies involving adolescents [13,15,24] and observed an increase in BMD in favor of football players (MD −0.02; CI 0.09 to 0.06; *p* = 0.66; I^2^ = 85%). The study that had the most weight (21.7%) was located in the second section [24]. No significant differences were observed between the groups of children and adolescents (Figure 3c).

##### Basketball vs. Swimming (Total Body and Upper and Lower Limbs)

- Total body

**Figure 4:**
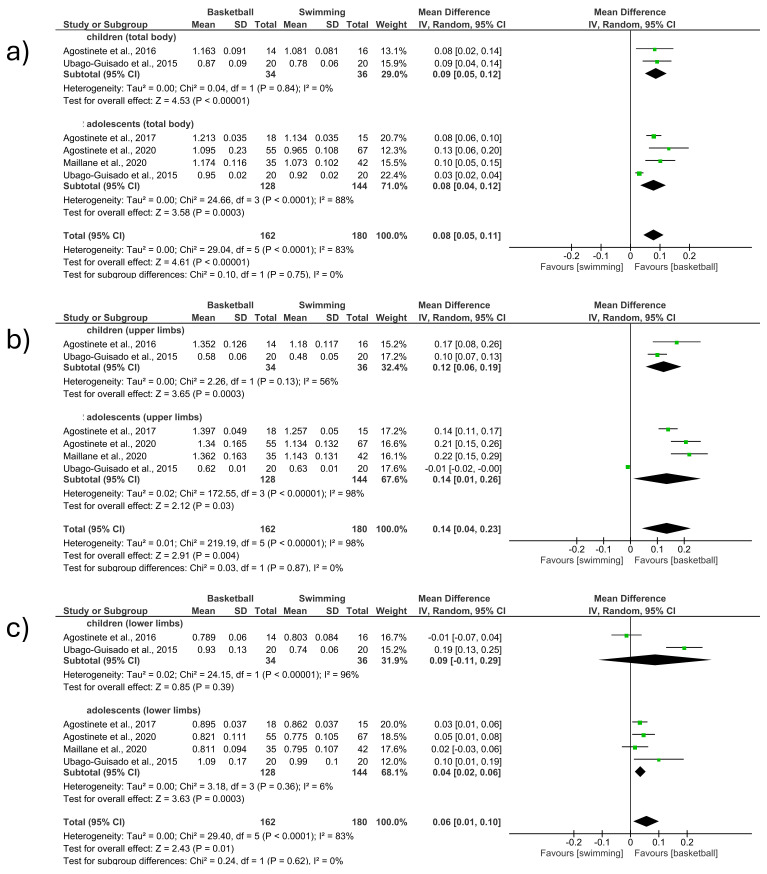
Effect of basketball interventions on BMD increase in children and adolescents compared to swimming interventions. (**a**) Forest plot illustrating the increase in total body BMD in favor of basketball. (**b**) Forest plot illustrating the increase in upper limb BMD in favor of basketball. (**c**) Forest plot illustrating the increase in lower limb BMD in favor of basketball [12,13,15,23,24].

The total body BMD was measured in five studies included in the meta-analysis, comparing basketball vs. swimming. (MD 0.08; CI 0.05 to 0.11; *p* < 0.001; I^2^ = 83%) (Figure 4a). The first section included studies involving children [12,15] and observed an increase in BMD in favor of basketball players (MD 0.09; CI 0.05 to 0.12; *p* < 0.001; I^2^ = 0%). The second section included studies involving adolescents [13,15,24,26] and observed an increase in BMD in favor of basketball players (MD 0.08; CI 0.04 to 0.12; *p* < 0.001; I^2^ = 88%). The study that had the most weight (22.4%) was located in the second section [15]. No significant differences were observed between the groups of children and adolescents (Figure 4a).

- Upper limbs

The upper limb BMD was measured in five studies included in the meta-analysis, comparing basketball vs. swimming. (MD 0.14; CI 0.04 to 0.23; *p* = 0.004; I^2^ = 98%) (Figure 4b). The first section included studies involving children [12,15] and observed an increase in BMD in favor of basketball players (MD 0.12; CI 0.06 to 0.19; *p* < 0.001; I^2^ = 56%). The second section included studies involving adolescents [13,15,24,26] and observed an increase in BMD in favor of basketball players (MD 0.14; CI 0.01 to 0.26; *p* = 0.03; I^2^ = 98%). The study that had the most weight (17.6%) was located in the second section [15]. No significant differences were observed between the groups of children and adolescents (Figure 4b).

- Lower limbs

The lower limb BMD was measured in five studies included in the meta-analysis, comparing basketball vs. swimming. (MD 0.06; CI 0.01 to 0.10; *p* = 0.01; I^2^ = 83%) (Figure 4c). The first section included studies involving children [12,15] and observed an increase in BMD in favor of basketball players (MD 0.09; CI −0.11 to 0.29; *p* = 0.39; I^2^ = 96%). The second section included studies involving adolescents [13,15,24,26] and observed an increase in BMD in favor of basketball players (MD 0.04; CI 0.02 to 0.06; *p* < 0.001; I^2^ = 6%). The study that had the most weight (20%) was located in the second section [23]. No significant differences were observed between the groups of children and adolescents (Figure 4c).

##### Basketball vs. Volleyball (Total Body)

- Total body

**Figure 5:**
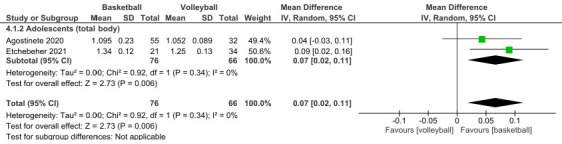
Effect of basketball interventions on BMD increase in adolescents compared to volleyball interventions. Forest plot illustrating the increase in total body BMD in favor of basketball. CI, confidence interval. SD, standard deviation [24,25].

The total body BMD was measured in two studies included in the meta-analysis, comparing basketball vs. volleyball. (MD 0.07; CI 0.02 to 0.11; *p* = 0.006; I^2^ = 0%) (Figure 5) [24,25]. The study that had the most weight (50.6%) was Etchebeher et al. [25]. All participants who played volleyball were adolescents (Figure 5).

##### Basketball vs. Judo (Total Body and Upper and Lower Limbs)

- Total body

**Figure 6:**
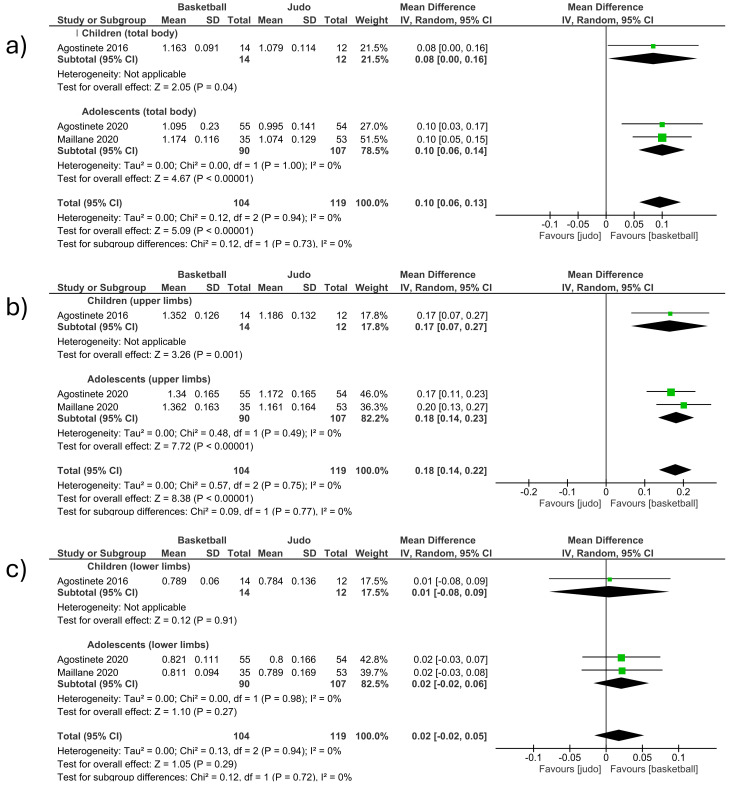
Effect of basketball interventions on BMD increase in children and adolescents compared to judo interventions. (**a**) Forest plot illustrating the increase in total body BMD in favor of basketball. (**b**) Forest plot illustrating the increase in upper limb BMD in favor of basketball. (**c**) Forest plot illustrating the increase in lower limb BMD in favor of basketball [12,13,24].

The total body BMD was measured in three studies included in the meta-analysis, comparing basketball vs. judo. (MD 0.10; CI 0.06 to 0.13; *p* < 0.001; I^2^ = 0%) (Figure 6a). The first section included studies involving children [12] and observed an increase in BMD in favor of basketball players. The second section included studies involving adolescents [13,24] and observed an increase in BMD in favor of basketball players. The study that had the most weight (27%) was located in the second section [24]. No significant differences were observed between the groups of children and adolescents (Figure 6a).

- Upper limbs

The upper limb BMD was measured in three studies included in the meta-analysis, comparing basketball vs. judo (MD 0.18; CI 0.14 to 0.22; *p* < 0.001; I^2^ = 0%) (Figure 6b). The first section included studies involving children [12] and observed an increase in BMD in favor of basketball players. The second section included studies involving adolescents [13,24] and observed an increase in BMD in favor of basketball players. The study that had the most weight (46%) was located in the second section [24]. No significant differences were observed between the groups of children and adolescents (Figure 6b).

- Lower limbs

The lower limb BMD was measured in three studies included in the meta-analysis, comparing basketball vs. judo. (MD 0.02; CI −0.02 to 0.05; *p* = 0.29; I^2^ = 0%) (Figure 6c). The first section included studies involving children [12] and observed an increase in BMD in favor of basketball players. The second section included studies involving adolescents [13,24] and observed an increase in BMD in favor of basketball players. The study that had the most weight (42.8%) was located in the second section [24]. No significant differences were observed between the groups of children and adolescents (Figure 6c).

##### Basketball vs. Karate (Total Body and Upper and Lower Limbs)

- Total body

**Figure 7:**
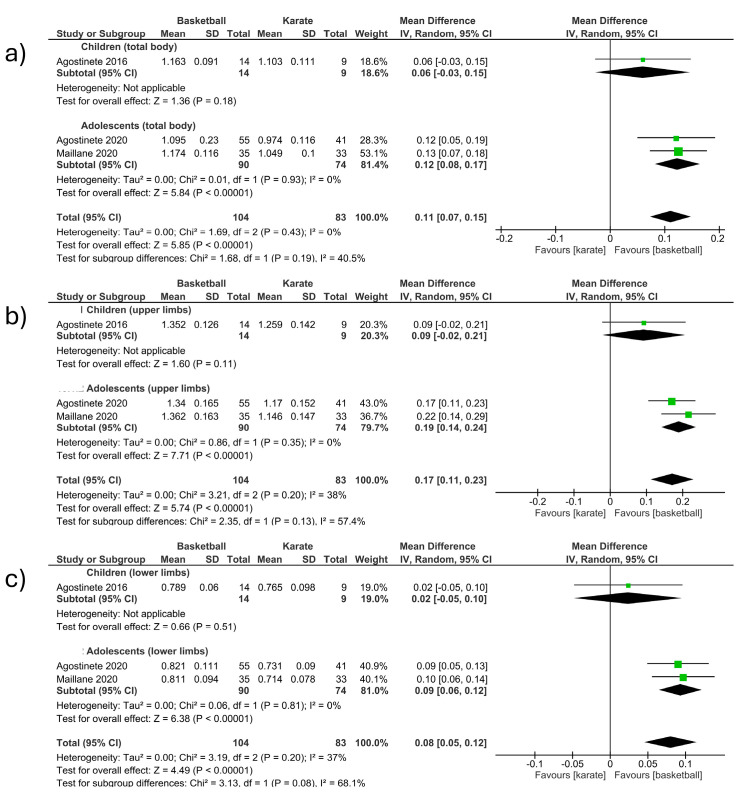
Effect of basketball interventions on BMD increase in children and adolescents compared to karate interventions. (**a**) Forest plot illustrating the increase in total body BMD in favor of basketball. (**b**) Forest plot illustrating the increase in upper limb BMD in favor of basketball. (**c**) Forest plot illustrating the increase in lower limb BMD in favor of basketball [12,13,24].

The total body BMD was measured in three studies included in the meta-analysis, comparing basketball vs. karate. (MD 0.11; CI 0.07 to 0.15; *p* < 0.001; I^2^ = 0%) (Figure 7a). The first section included studies involving children [12] and observed an increase in BMD in favor of basketball players. The second section included studies involving adolescents [13,24] and observed an increase in BMD in favor of basketball players. The study that had the most weight (53.1%) was located in the second section [13]. No significant differences were observed between the groups of children and adolescents (Figure 7a).

- Upper limbs

The upper limb BMD was measured in three studies included in the meta-analysis, comparing basketball vs. karate. (MD 0.17; CI 0.11 to 0.23 *p* < 0.001; I^2^ = 38%) (Figure 7b). The first section included studies involving children [12] and observed an increase in BMD in favor of basketball players. The second section included studies involving adolescents [13,24] and observed an increase in BMD in favor of basketball players. The study that had the most weight (43%) was located in the second section [24]. No significant differences were observed between the groups of children and adolescents (Figure 7b).

- Lower limbs

The lower limb BMD was measured in three studies included in the meta-analysis, comparing basketball vs. karate. (MD 0.08; CI 0.05 to 0.12; *p* < 0.001; I^2^ = 37%) (Figure 7c). The first section included studies involving children [12] and observed an increase in BMD in favor of basketball players. The second section included studies involving adolescents [13,24] and observed an increase in BMD in favor of basketball players. The study that had the most weight (40.9%) was located in the second section [24]. No significant differences were observed between the groups of children and adolescents (Figure 7c).

##### Basketball vs. Kung Fu (Total Body and Upper and Lower Limbs)

- Total body

**Figure 8:**
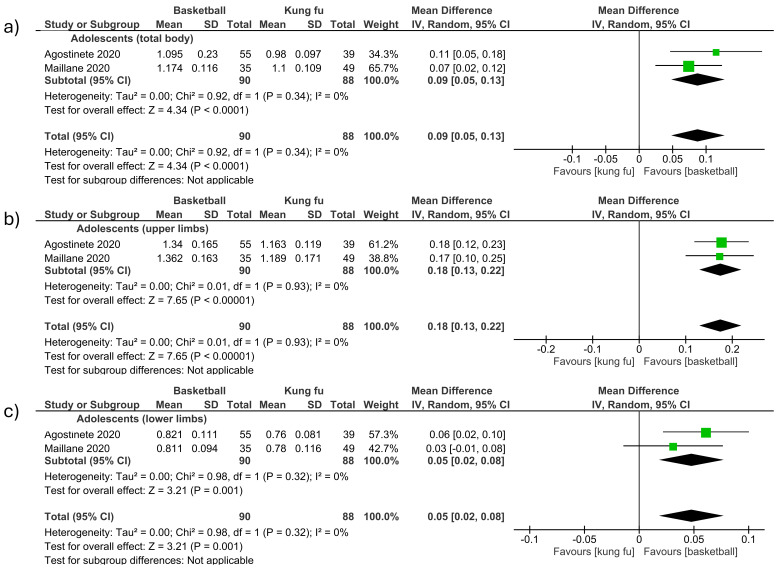
Effect of basketball interventions on BMD increase in children and adolescents compared to kung fu interventions. (**a**) Forest plot illustrating the increase in total body BMD in favor of basketball. (**b**) Forest plot illustrating the increase in upper limb BMD in favor of basketball. (**c**) Forest plot illustrating the increase in lower limb BMD in favor of basketball [13,24].

The total body BMD was measured in two studies included in the meta-analysis, comparing basketball vs. judo in adolescents (MD 0.09; CI 0.05 to 0.13; *p* < 0.001; I^2^ = 0%) (Figure 8a).

- Upper limbs

The upper limb BMD was measured in two studies included in the meta-analysis, comparing basketball vs. judo in adolescents (MD 0.18; CI 0.13 to 0.22; *p* < 0.001; I^2^ = 0%) (Figure 8b).

- Lower limbs

The lower limb BMD was measured in two studies included in the meta-analysis, comparing basketball vs. judo in adolescents (MD 0.05; CI 0.02 to 0.08; *p* = 0.001; I^2^ = 0%) (Figure 8c).

## 4. Discussion

This meta-analysis compared BMD among children and adolescents engaged in basketball (considered a high-impact sport), those involved in various other sports (with differing impact status), and those who engaged in free activity. The findings indicated that children and adolescents participating in basketball seem to exhibit higher BMD (upper and lower limbs) compared to other sports and free activity.

### 4.1. Basketball vs. Free Activity

Comparing the basketball group to the free activity group, significant results favoring basketball were obtained for upper limbs, lower limbs, and total body, although a high heterogeneity related to the significance and methodological diversity across the studies was observed. The study that exerted the most weight in this meta-analysis did so due to the training duration. This study involved a cohort of female adolescents engaging in an average training duration of 360 min per week [15]. Findings suggest that participation in basketball during youth may offer substantial advantages in bone health. This is attributed to the high-impact nature and specific physical demands of the sport, such as explosive movements and repeated mechanical loads [14]. Unlike sedentary individuals who lack this physical stimulus, the constant stimulation of the musculoskeletal system during training and competition in sports can promote a notable improvement in BMD in children and adolescents [7,27]. Therefore, the research findings suggest that not practicing sports or engaging in free activity in children and adolescents may not favor the development of BMD [29,30].

### 4.2. Basketball and Sports Focusing on Lower Limbs (Football and Athletics)

The majority of studies found higher BMD in sports focusing on upper limbs compared to basketball. Various studies [31,32,33] demonstrated that basketball players exhibited higher BMD than football players, attributed to the higher frequency of jumps, intermittent runs, abrupt braking, and sprints intrinsic to basketball. In contrast, football involved more continuous and repetitive runs, resulting in comparatively lower BMD. The varying loading patterns between these sports underscore the higher BMD associated with activities like basketball that encompass diverse movement patterns compared to sports lacking such variability [15]. Upon individual analysis of the lower limbs, football displays a more developed BMD compared to basketball, presenting a notable discrepancy. General assessments of BMD across the total body, as seen previously, favor basketball. However, when specifically scrutinizing the lower and upper limbs, football and athletics demonstrate greater benefits in BMD. This divergence could be attributed to an emphasis on strengthening the lower limbs, which are extensively utilized in sports. As football predominantly engages these muscle groups, focused attention on the lower limbs may result in comparatively more developed lower limb BMD in football players compared to basketball players [31]. However, delving further into this aspect necessitates additional research specifically comparing these two sports. When measuring BMD, similar results are obtained to those obtained when measuring it in various parts of the body.

### 4.3. Basketball vs. Swimming

This meta-analysis compared BMD between basketball players and swimmers, finding higher BMD in the basketball group. Basketball players tend to develop relatively higher BMD compared to those who engage in non-impact sports such as swimming [34]. Across all studies included, the basketball group consistently exhibited greater BMD for the total body. However, in the upper limbs, one study involving female participants reported lower BMD in the basketball group [15]. Likewise, in the lower limbs, a study of children-aged boys demonstrated superior BMD in the swimmer group [12]. These discrepancies may be attributed to the potential influence of these participants’ lifestyles. The study that carried the most weight was conducted in adolescent girls [15]. Swimming, categorized as a non-impact sport, occurs in a hypo-gravity setting, requiring minimal force from muscular contractions against gravity [35]. Consequently, this reduced stress on the bones leads to comparatively lower BMD status compared to basketball [36].

### 4.4. Basketball and Sport with Emphasis on Total Body (Handball, Baseball, Gymnastics, and Volleyball)

Basketball showed a greater effect on BMD compared to sport with emphasis on total body, but not uniformly across all cases. A study involving handball training showed higher BMD than the basketball group in total body [15]. Handball players are more focused on running, passing, dribbling, and shooting the ball, with more emphasis on jumps and abrupt changes in direction [37]. Handball players are more exposed to collisions and physical contact with opponents, which could influence the distribution of mechanical load on the body and bone response [37].

In baseball, movements are more specific and focused on technical skills, such as throwing with precision, hitting the ball with the bat, and running from base to base, unlike basketball [38].

When comparing gymnastics to basketball, it is evident that basketball involves high impact during gameplay, potentially increasing the load and stress on the bones [13]. In contrast, in gymnastics, although demanding and frequently acrobatic movements are performed, which may promote BMD development, the impact is lower, particularly in events such as individual artistic gymnastics [39].

Analyzing the resemblance between basketball and volleyball, numerous parallels exist, such as the frequency of jumps resulting in considerable ground impacts. However, subtle differences contribute to higher BMD status among basketball players compared to volleyball players [40,41]. In volleyball there is a 6-s rally time followed by approximately 14 s of rest, where players do not move from a specific space on the court [40], whereas, in basketball, high-intensity movements occur more frequently, exerting increased stress on the skeletal system, consequently contributing to an elevated BMD [41].

### 4.5. Basketball vs. Combat Sports (Karate, Judo, and Kung Fu)

In comparison between combat sports and basketball, the basketball group consistently demonstrated higher BMD across all categories, including upper limbs, lower limbs, and total body. Additionally, within combat sports, judo and kung fu showcased the highest BMD levels, attributed to the training complexity associated with these disciplines compared to karate [42]. In combat sports such as karate, judo, and kung fu, where specific technical movements and strength and endurance exercises are performed, the nature of impacts can differ from basketball [43]. While strikes, kicks, throws, and controlled falls are experienced, they may not necessarily generate the same consistent and repetitive mechanical load on the musculoskeletal system as seen in basketball [44]. Furthermore, strikes and kicks in combat sports may target specific areas of the body, whereas in basketball, the mechanical load is more evenly distributed throughout the body due to the dynamic nature of the game [45].

### 4.6. Sex-Based Disparities in BMD

One of the studies included in this section was conducted with girls, which revealed some differences in the results, as girls exhibited lower BMD than studies involving male teams [15]. A recent statement from the International Osteoporosis Foundation suggests that girls have a lower rate of bone accrual than boys, and this difference is even more pronounced at the onset of puberty [6]. The differences observed between sexes across different limbs can be attributed to various reasons. For the upper limbs, it is possible that, under normal conditions, boys have more muscle mass than girls, as is also the case for the lower limbs, resulting in girls being slower than boys. In the case of a study included in our meta-analysis conducted on girls [15], these girls have not yet reached the age at which bone mineralization typically increases, which is usually between 11 and 15 years of age [46]. Additionally, analyzing their body weights, it is possible that they do not have sufficient weight to impose tension on the skeletal system that would stimulate BMD accumulation. Research indicates that men typically weigh more than girls, and therefore, their impact may have a greater effect on their BMD [13]. According to a study, boys have larger and stronger bone and joint surfaces and more bone at muscle attachment sites, while females have stronger pelvises due to their ability to carry a child and experience childbirth [47]. Additionally, differences in testosterone and estrogen levels contribute to the discrepancy in bone mass between boys and girls, with testosterone promoting bone size in boys and estrogen reducing bone growth while regulating bone mineral levels in girls [15,48]. Moreover, these hormonal differences not only influence bone size and mineralization but also affect bone geometry and strength [49]. In boys, the anabolic effects of testosterone promote greater cortical bone thickness and a higher bone strength index by enhancing periosteal apposition [50]. In contrast, estrogen in girls stimulates endosteal bone formation while limiting excessive periosteal expansion, leading to a more compact but narrower bone structure [50]. These sex-specific differences contribute to variations in fracture risk, with boys typically experiencing more trauma-related fractures during adolescence, whereas girls face a higher risk of osteoporosis and fragility fractures later in life due to declining estrogen levels after menopause [51]. A study found that female adolescents accumulated spinal bone mineral more rapidly than boys, but longitudinal studies did not find gender differences in bone mass development. However, another study reported that boys had higher femoral bone mass, while girls had higher spinal bone mass, which could be attributed to a higher number of boys compared to girls in the study [49].

### 4.7. Limitations and Future Research

Limitations were identified in the study selection process, primarily due to the limited availability of studies offering detailed data breakdowns for specific body segments, such as the lower limbs, upper limbs, and total body measurements. Additionally, several studies were excluded from the meta-analysis due to inaccuracies in the collection of BMD data, including unspecified measurement methods and missing values. Furthermore, inadequate descriptions of the type of training conducted by athletes and the weekly time commitment to sports were noted in some cases.

There is very little literature on randomized controlled studies measuring BMD in children, in which an intervention is carried out by comparing basketball with other sports or not practicing any sport, and therefore, it has been necessary to select mainly observational studies.

The heterogeneity found in this meta-analysis is notably high, which is understandable, given that different sports have been compared. To assess the robustness of the results and the impact of the included studies, a sensitivity analysis was performed. This involved the sequential exclusion of individual studies and re-evaluation of the effect on all outcome variables whenever heterogeneity in the results was moderate to high. However, despite these efforts, heterogeneity remained high, and a sensitivity analysis could not be performed beyond conducting an analysis of the subgroups of children and adolescents. Therefore, the results presented in this meta-analysis should be interpreted with caution. This heterogeneity can significantly contribute to the variability observed in the results. It is important to recognize that the training in different studies involves a variety of movements, intensities, and loads on the musculoskeletal system, which can influence bone response heterogeneously among the study groups. Given that only a limited number of studies have examined the importance of training frequency on BMD, future research should further investigate its role in maximizing BMD gains while also determining the optimal intensity and volume for bone health benefits. Additionally, monitoring vitamin D status could offer valuable insights into its importance in exercise and, therefore, its contribution to bone mineralization. A comprehensive approach that integrates exercise diversity, training load, and nutritional factors will be crucial for developing more effective strategies to enhance bone health during growth

## 5. Conclusions

It appears that basketball stands out as one of the most effective sports for enhancing BMD in children and adolescents when compared to football, swimming, combat sports, and other team sports like baseball, volleyball, athletics, and gymnastics. Given the crucial role of this developmental phase in achieving optimal BMD for future health, basketball is highly recommended for children and adolescents aiming to maximize bone mass. While other sports also positively influence BMD, basketball stands out as the most effective option for enhancing it.

## Figures and Tables

**Table 1:** Characteristics of the included studies assessing the effects of basketball on BMD compared to other sports and free activity in children and adolescents.

Article (Author and Age)	Country	Age (Years)	Sex	Sport Group	Sample	Variables	Practice Time (Months)	Training Frequency (Minutes per Week)
Agostinete et al., 2016 [12]	Brazil	11.9 (1.85)	M	Basketball	14	Upper limbs	Minimum 6 months Free activity: non-sport in the last 3 months	The information is not provided in the article
				Karate	9	Lower limbs		
				Football	18	Spinal column		
				Judo	12	Total body		
				Swimming	16			
	
				Free activity	13			
Agostinete et al., 2017 [23]	Brazil	14.34 (0.26)	M	Swimming	15	Upper limbs	The information is not provided in the article	Average of 1108 min per week
				Basketball	18	Lower limbs		
						Trunk		
				Free activity	20	Total body		
Agostinete et al., 2020 [24]	Brazil	14.1 (1.91)	M	Basketball	55	Upper limbs	Minimum 6 months	Minutes per week:
				Football	100	Lower limbs		
				Swimming	67	Spinal column		Basketball: 182.9
				Volleyball	32	Total body		Football: 163.3
				Karate	41			Swimming: 164
				Judo	54			Volleyball: 121.9
				Kung fu	39			Karate: 139
				Baseball	22			Judo: 104.2
				Gymnastics	16			Kung fu: 106.4
				Athletics	27			Baseball: 191.4
								Gymnastics: 206.3
				Free activity	172			Athletics: 161.1
Etchebehere et al., 2021 [25]	Brazil	16.7 (0.92)	M	Basketball	21	Total body	36 months	900 min per week
				Volleyball	34			
Jallai et al., 2017 [9]	Estonia	Basketball	M	Basketball	12	Total body	An average of 8 (±2.9) years of sporting activity	Minutes per week: Basketball: 558 Football: 546
		16.3 (0.7)				Head		
		Football		Football	15	Lumbar spine		
		16 (0.3)				Right arm		
						Left arm		
						Right leg		
						Left leg		
						Right femur		
						Left femur		
						F neck D		
						F neck I		
						F Trochanter D		
						F Trochanter I		
Junior et al., 2017 [26]	Brazil	Basketball 13.4 (1.2) Free activity 11.9 (2.2)	M	Basketball Free activity	14 13	Upper limbs	Minimum 6 months Free activity: non-sport in the last 3 months	The information is not provided in the article
						Lower limbs		
						Total body		
						Spinal column		
Maillane et al., 2020 [13]	Brazil	13.3 (1.7)	M	Swimming	42	Upper limbs Lower limbs Total body	Minimum 6 months	The information is not provided in the article
				Football	106			
				Karate	33			
				Judo	53			
				Kung fu	49			
				Basketball	35			
	
				Free activity	111			
Ubago-Guisado et al., 2015 (prepub and pub) [15]	Spain	Prepub: 9.16 (0.69); Pub: 12.20 (0.62)	F	Basketball	20	Total body Upper limbs Lower limbs	Minimum 8 months	360 min per week
				Soccer	20			
				Handball	20			
	
				Free activity	20			
Zribi et al., 2014 [27]	Tunisia	Basketball 11.1 (0.8) Free activity 11.2 (0.7)	M	Basketball	25	Total body	Minimum 12 months	180 min per week
						Leg dominant		
						L2–L4		
						Femoral neck		
						Trochanter		
						Total hip		
				Free activity	26			

(pub): puberal children; (prepub): prepuberal children; F: female; M: male.

## Data Availability

The data presented in this study are available upon reasonable request from the corresponding author.

## Supplementary Materials

The following supporting information can be downloaded at: https://www.mdpi.com/article/10.3390/children12030271/s1, Table S1: PRISMA Checklist; File S1: Search strategy; File S2: Funnel plot; Table S2: reason for exclusion table; Figure S1: Risk of bias.

## Abbreviations

The following abbreviations are used in this manuscript:

DOAJ	Directory of open access journals
TLA	Three letter acronym
LD	Linear dichroism
